# To Regulate or Not to Regulate: Emotion Regulation in Participants With Low and High Impulsivity

**DOI:** 10.3389/fnbeh.2021.645052

**Published:** 2021-07-30

**Authors:** Moritz Julian Maier, Julian Elias Schiel, David Rosenbaum, Martin Hautzinger, Andreas Jochen Fallgatter, Ann-Christine Ehlis

**Affiliations:** ^1^Center for Responsible Research and Innovation, Fraunhofer Institute for Industrial Engineering, Berlin, Germany; ^2^Department of Psychiatry and Psychotherapy, Faculty of Medicine, Medical Center – University of Freiburg, Freiburg, Germany; ^3^Department of Psychiatry and Psychotherapy, Tübingen Center for Mental Health (TüCMH), University of Tübingen, Tübingen, Germany; ^4^Department of Psychology, Eberhard Karls University, Tübingen, Germany

**Keywords:** fNIRS, DLPFC, cognitive control network, cognitive control, impulsivity

## Abstract

Successful emotion regulation plays a key role in psychological health and well-being. This study examines (1) whether cognitive control and corresponding neural connectivity are associated with emotion regulation and (2) to what extent external instructions can improve emotion regulation in individuals with low vs. high cognitive control capacity. For this, emotion regulation capabilities and the impact of emotion regulation on a subsequent emotional Stroop task was tested in participants with low (*N* = 25) vs. high impulsivity (*N* = 32). The classification according to impulsivity is based upon the stable correlation between high impulsivity and reduced cognitive control capacity. A negative emotion inducing movie scene was presented with the instruction to either suppress or allow all emotions that arose. This was followed by an emotional Stroop task. Electromyography (EMG) over the corrugator supercilii was used to assess the effects of emotion regulation. Neurophysiological mechanisms were measured using functional near-infrared spectroscopy over frontal brain areas. While EMG activation was low in the low-impulsive group independent of instruction, high-impulsive participants showed increased EMG activity when they were not explicitly instructed to suppress arising emotions. Given the same extent of functional connectivity within frontal lobe networks, the low-impulsive participants controlled their emotions better (less EMG activation) than the high-impulsive participants. In the Stroop task, the low-impulsive subjects performed significantly better. The emotion regulation condition had no significant effect on the results. We conclude that the cognitive control network is closely associated with emotion regulation capabilities. Individuals with high cognitive control show implicit capabilities for emotion regulation. Individuals with low cognitive control require external instructions (= explicit emotion regulation) to achieve similarly low expressions of emotionality. Implications for clinical applications aiming to improve emotion regulation are discussed.

## Introduction

Conscious cognitive control is often perceived as a key element of a “desirable life” (cf. Inzlicht et al., 2015) and in this regard associated with academic success (Nota et al., 2004), physical health, reduced substance dependence, better personal finances, and less criminal offenses (Moffitt et al., 2011). Cognitive control is commonly seen as a constitutive resource on which all higher functions (e.g., mental set shifting, updating and monitoring, and inhibition of prepotent responses; Miyake et al., 2000) are built (e.g., Miller and Cohen, 2001). When applying cognitive control in an affective context, the construct of emotion regulation must additionally be considered. This raises the question of whether there is a correlation between cognitive control and the capability to deal with (negative) emotions in a functional (adaptive) way. For the current study, this correlation is of particular interest. Ochsner and Gross (2005) described a wide range of possible targets for effects of cognitive control on emotion, ranging from basal attentional processes to cognitive appraisal and reappraisal. The mechanisms of cognitive change and their neural correlates are particularly frequently mentioned in studies investigating cognitive control and emotion regulation. Summarizing previous findings, Ochsner and Gross (2005) differentiate between two types of control processes: the direct type and the indirect type. Whereas the direct type relies on a reciprocal connection of the ventral PFC (VPFC) and the orbitofrontal cortex (OFC) with subcortical emotional appraisal systems (e.g., amygdala), the indirect type involves the DLPFC and is assumed to influence appraisal systems only mediately (e.g., via VPFC). With respect to these neurophysiological considerations, Ochsner et al. (2012) outlined a cognitive model describing the multifaceted influence of cognitive control on emotion. The proposed model encompasses a broad spectrum of targets for cognitive control, ranging from rather proactive influence (situation selection and modification, attentional deployment) to rather reactive influence (cognitive change, response modulation) on emotion. For the paradigm of the current study (emotion induction using a short film clip), internal situation modification, attentional processes, appraisal and reappraisal, as well as response modulation are of particular interest. The PFC and especially the DLPFC (explicit appraisal processes, Ochsner and Gross, 2005; selective attention and working memory, Ochsner et al., 2012) are considered to play a crucial role for these processes.

Aiming to establish a comprehensive model of cognitive control within the PFC, Ridderinkhof et al. (2004) unraveled the role of different PFC substructures in different control processes. Their key conclusion is that cognitive control can be divided into two main stages: detecting errors and conflicting response tendencies, which is associated with the medial frontal cortex (MFC); and implementing appropriate adjustments, which is associated with lateral and orbitofrontal divisions of the PFC. The rostral cingulate zone (RCZ, border zone between BA8, BA6, BA32’, and BA24’) constitutes an important link between these two stages. In particular, interconnectivity between anterior cingulate cortex (ACC; BA 24, BA24’, BA32’) and DLPFC (BA46) areas (Koski and Paus, 2000) via the RCZ seems to play a crucial role for a cognitive control network (CCN) within the PFC. The idea of a superordinate CCN is also addressed by Niendam et al. (2012). In their meta-analysis, they gather evidence for connectivity patterns involving dorsolateral prefrontal, anterior cingulate, and parietal cortices. Since regulation processes can only be understood as a complex interplay of multiple neural structures, functional connectivity analyses have often been used to study the CCN in the past. Furthermore, functional connectivity analyses of Raz et al. (2016) support a domain-general network model that shows how emotions are represented at a neural level. Their results strengthen the assumption that there might be a common neural network for different emotions (e.g., Barrett, 2006). Raz et al. (2016) emphasize that, in addition to structures of the ventral stream, increased functional connectivity between dorsal and ventral structures plays a fundamental role during emotion induction. Referring to the neural model of Ochsner and Gross (2005), one could conclude, with some limitations, that the direct (ventral) type and the indirect (dorsal) type of control processes also interact considerably. A differentiated view of the interplay of distinct aspects of regulation processes might therefore help to identify factors determining success or failure of cognitive control (of emotion).

While CCN studies and meta-analyses have mainly taken a micro-analytic view of the PFC, connectivity studies investigating emotion regulation processes have taken a macro-analytic perspective on the interplay between cortical and subcortical structures. In this study, we aim to combine connectivity analysis with a differentiated view of *within-PFC-connectivity* in emotion regulation processes. In this regard, the role of the DLPFC and its substructure BA46 as an important link between the CCN components DLPFC and MFC (Ridderinkhof et al., 2004) is of particular interest. While most research to date has focused either on the influence of emotion on cognitive control (e.g., Gray and Braver, 2002) or on the influence of cognitive control on emotion (e.g., Ochsner and Gross, 2005), here we consider both directions. Since at least partially the same brain structures play a role for both directions of influence (e.g., the DLPFC), reciprocal interference seems very likely.

To address this question, we combined negative emotion induction, an emotional Stroop task, and a high- vs. low-impulsive sample with optical imaging of relevant PFC substructures (fNIRS = functional near-infrared spectroscopy) and electromyography (EMG) over the corrugator supercilii as an indicator of negative emotion (cf., Cacioppo et al., 1986; Lang et al., 1993). Considering the human face as a site of emotional expression, previous studies suggest a linear association between stimulus valence and EMG activity of emotion-specific facial muscles (e.g., between negative stimuli and the corrugator supercilii: Larsen et al., 2003). In detail, two subsamples (high- vs. low-impulsive participant groups) were compared regarding the interplay of cognitive control and emotion. Classification by impulsivity is based on the stable correlation between impulsivity and aspects of cognitive control (e.g., Logan et al., 1997; Herrmann et al., 2010), with high impulsivity associated with reduced cognitive control capacity. During the experiment, each person underwent negative emotion induction after being instructed to either suppress or allow arising emotions. At this point, the influence of cognitive control (high vs. low) on the downregulation of negative emotions can be observed in EMG data. With the implementation of different instructions as a between-participants factor, two things can be assessed: First, the ability of high- vs. low-impulsive participants to regulate their emotions, and second, the influence of emotion regulation on the subsequent task performance. In a next step, each participant had to complete a modified emotional Stroop task that required cognitive control to overcome an emotion-based cognitive conflict. At this point, the influence of emotion on cognitive control performance becomes apparent. Regarding underlying neurophysiological correlates, a closer look at the interplay of PFC substructures within the CCN using functional connectivity analyses is particularly interesting. Considering previous research and established assumptions as presented above, we suggest the following hypotheses:

High-impulsive participants show more muscle contraction of the corrugator supercilii, decreased connectivity within the cognitive control region DLPFC, and poorer performance in the emotional Stroop task in comparison to persons of the low-impulsive group. Given that, according to Gray and Braver (2002), negative emotions aggravate cognitive control performance on verbal stimuli, we expect participants to show increased DLPFC activation and a better performance in the subsequent emotional Stroop task when emotion induction occurred with proactive suppression compared to the “allow all upcoming feelings” condition. In light of findings that cognitive control is involved in emotion processing in general (Ochsner and Gross, 2005) and in downregulating negative emotions in particular (Ochsner et al., 2012), we expect that proactive suppression of emotions during emotion induction is less effectual for the low-impulsive group than for the high-impulsive group (= interaction effect of group and instruction on EMG activation, connectivity patterns and emotional Stroop task performance). Regarding the connectivity data and the CCN, we hypothesize that the interaction between PFC structures is significantly increased for low-impulsive vs. high-impulsive participants and for the suppression vs. allowance instruction.

## Materials and Methods

### Participants

The original sample consisted of 61 participants. Four persons dropped out due to erroneous motion and bite artifacts or technical issues. The composition of the final sample (*N* = 57) is depicted in Table 1. The mean age was 22.8 years (*SD* = 2.8). All participants were students at the University of Tuebingen (Germany). Level of impulsivity was measured using the Adult ADHD Self-Report Scale (ASRS), with participants with scores < 10 classified as “low-impulsive” and participants with scores between 15 and 23 classified as “high-impulsive”. Potential study participants with ASRS scores on the online screening questionnaire that indicated ADHD (scores higher than 23), other psychiatric or neurological diseases or medication consumption (with the exception of the contraceptive pill) were not invited, and the data were immediately deleted for data protection reasons. While 25 participants were assigned to the low-impulsive group (mean age = 22.8 years, *SD* = 3.0), 32 participants were assigned to the high-impulsive group (mean age = 22.8 years, *SD* = 2.7). To avoid comorbidities commonly associated with ADHD, participants with high impulsivity, but without an ADHD diagnosis and with ASRS scores not exceeding 23, were selected for the high-impulsive group. In terms of gender ratio and age, the groups did not differ significantly (see Table 1). All participants received either money (10 € per hour) or course credits for compensation. The ethics committee of the University Hospital and the University of Tuebingen approved this project and all participants gave written informed consent. All methods and procedures used in this study were in accordance with the current guidelines of the World Medical Association Declaration of Helsinki (General Assembly of the World Medical Association, 2014).

**TABLE 1:** Sample characteristics: fNIRS data, number of females and males as a function of subsample (high- vs. low-impulsive) and instruction (suppress vs. allow emotions), and chi-squared test of subgroup sex ratio.

		Low-impulsive Group (*N* = 25)		High-impulsive Group (*N* = 32)	
Instruction	Chi-squared	Female	Male	Female	Male
Suppress (*N* = 29)	χ^2^(1) = 1.04, *p* = 0.307	12	1	13	3
Allow (*N* = 28)	χ^2^(1) = 1.78, *p* = 0.182	11	1	12	4

*Randomized but balanced assignment of instructions.*

### Questionnaires

The ASRS (Mörstedt et al., 2016) measures impulsivity and attention deficits with 18 items and a scale from 0 to 5. Regarding the quality criteria of the ASRS, the internal consistency ranges between 0.63 and 0.72; the test-retest reliability between 0.58 and 0.77. Furthermore the ASRS shows a high predicitive validity for a clinical diagnosis (AUC = 0.90) (Kessler et al., 2007; Hines et al., 2012), while maintaining construct-, factorial- and criterion validity (Lauth and Minsel, 2014; Carlucci et al., 2017). Additionally, participants had to report if they experienced anger, fear or sadness during the emotion induction paradigm immediately after watching the film clip (see below). Each of the three emotions had to be rated on a 1–5 Likert scale [“Did you experience anger/fear/sadness during the film clip?”—(1) “very little,” (2) “little,” (3) “moderately,” (4) “much,” (5) “very much”]. Results depending on group and instructions are listed in Supplementary Table 1.

### Justification of Sample Size

Effect sizes from the Marsh et al. (2002) study were used to determine the sample size, using GPower (Faul et al., 2009). The effect size ranged from 0.19 to 0.27 Cohen’s α.

### Design

This study was a 2 (low vs. high impulsive) × 2 (instruction allow vs. suppress arising emotions) between-participants design with one measurement per participant. This design, in which each participant was instructed one way or another, was chosen to avoid carryover effects.

### Emotion Induction Paradigm

Negative emotions were induced in all participants with a 4-min film clip. Stereo sound was realized with two standard PC speakers positioned on both sides of the monitor (standardized volume across all participants, peaks approximately 80 dB). EMG and fNIRS were prepared before watching the movie and measured during the movie scene. The footage shown was from the movie Sophie’s Choice (Pakula, 1982) and has been successfully used for emotion induction in previous studies (e.g., Fitzgerald et al., 2011; Möbius et al., 2017). Editing was performed using Microsoft’s Windows Movie Maker software. The scene presented shows a sadistic concentration camp guard forcing a Polish woman to decide which of her two children is to be killed. The wording of the previous instruction (allow vs. suppress arising emotions) was adapted from Gross (1998) and Hayes et al. (2010). Participants were instructed to “suppress arising feelings as if they watched the clip in a situation where an emotional reaction is unwanted.” They were not given a strategy to achieve this.

### Emotional Stroop Task

**FIGURE 1:**
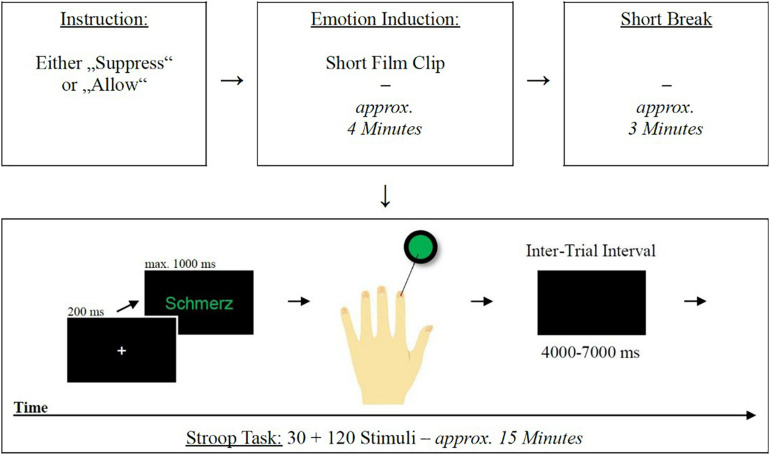
Schematic representation of the experimental procedures. Emotional Stroop task procedure (bottom): from left to right—(1) fixation cross, (2) stimulus presentation, (3) response (in the case of a stimulus with green font color, participants had to respond with their left index finger), (4) ITI, (5) begin of next trial.

After the emotion induction, with a short break of approximately 3 min, the emotional Stroop task started (Watts et al., 1986). The task consisted of word lists of 10 stimuli each with negative, positive and neutral valence (based on stimuli by Smith and Waterman, 2003). The 30 words were presented in 4 different colors (red, green, blue, and yellow) centrally against a black background. This resulted in 120 different stimuli. Responses were given by means of a high-frequency button box (The Black Box ToolKit Ltd., United Kingdom) with four buttons (one for each color), allowing precise recording of reaction times (see Supplementary Figure 3). A button-color assignment was displayed throughout the experiment. After 20 training trials and a fNIRS baseline scan (20 s), the experiment started with a white fixation cross (200 ms) followed by a target stimulus remaining on the screen until a response was given (timeout after 1,000 ms). In case of an incorrect button press, no error message appeared. Between trials, a black screen appeared for a jittered (Plichta et al., 2007) period of 4,000–7,000 ms. Figure 1 illustrates the integration of the mentioned components in the course of the experiment.

### EMG

A BrainAmpExG (Brain Products GmbH, Gilching, Germany) MR 16-channel system amplifier was used to record EMG. Two EMG electrodes were placed over the left corrugator supercilii. Vertical (VEOG) and orthogonal electrooculography (OEOG) were additionally applied for correcting the EMG data. Fz was used as the ground according to the international 10–20 system (Jasper, 1958). The sampling rate was 1,000 Hz. An online cutoff filter for data < 0.1 Hz and > 70 Hz and a notch filter of 50 Hz were applied.

### EMG Analyses

A total of 13 participants were excluded from the EMG analysis (*N* = 48). Four cases were hardware malfunctions, three were software malfunctions, and six were outliers (defined as two standard deviations over/below the overall mean standard deviation). Exclusions were evenly distributed over all groups. All analyses were run using Brain Vision Analyzer (Brain Products GmbH, Gilching, Germany). Preprocessing of the EMG data was adapted from Elkins-Brown et al. (2016). Blink artifacts were corrected via automatic ocular correction. An IIR bandpass filter (28–499 Hz) and a 50 Hz notch filter were applied with an additional moving average correction (20 ms). Afterward, the data were split into 12 segments of 20 seconds, a Fast Fourier Transform was applied, and a mean was calculated for each segment. In accordance with related works (e.g., Van Boxtel, 2001, 2010), the mean of the spectrum between 60 and 85 Hz was exported separately for each participant and each time point. SPSS 22 (SPSS Inc., Chicago, United States) was used to perform a 2 × 2 × 12 ANOVA for repeated measurements with the between-participants factors of instruction and group and the within-participants factor of time. For *post-hoc* analysis, data were merged over all time points and paired *t*-tests were performed for both instruction conditions.

### fNIRS

Using fNIRS, a non-invasive optical imaging technique, *in vivo* measurement of changes in the concentration of oxygenated (O_2_Hb) and deoxygenated (HHb) hemoglobin in cortical brain tissue is possible. The ETG-4000 Optical Topography System (Hitachi Medical Co., Japan) was used to conduct the fNIRS measurements. This is a continuous wave system with two different wavelengths (695 ± 20 and 830 ± 20 nm) and a temporal resolution of up to 10 Hz. A 3 × 11 probe set with 52 channels, 16 detectors and 17 emitters, and an inter-optode distance of 3 cm was placed over the left and right frontopolar areas. In accordance with the international 10–20 system (Jasper, 1958), the medial optode was located in the bottom row on Fpz and symmetrically oriented toward T3/T4.

### fNIRS Data Preprocessing

Raw data from the fNIRS measurements was exported and analyses were performed with MATLAB (2015b) (The MathWorks, Natick, MA, United States). All frequencies < 0.01 Hz and > 0.5 Hz were excluded using a bandpass filter. Additionally, a correlation-based signal improvement (CBSI; Cui et al., 2010) procedure was applied to correct motion artifacts. All further analyses were run with the calculated cbsi-hb. Independent Component Analysis (ICA; Delorme and Makeig, 2004) was used to exclude high-amplitude artifacts. Thereafter, all signals were visually inspected for remaining artifacts after the described preprocessing. In case of visible artifacts, the channels were interpolated from surrounding channels. Subsequently, the mean activation in the different regions of interest (ROI) was exported for all further analyses. As they are part of the CCN, the following ROIs were exported: left and right hemispheric Brodmann area 9 (part of the frontal cortex contributing to the dorsolateral and medial prefrontal cortex), area 10 (anterior-most portion of the prefrontal cortex) and area 46, as well as the inferior frontal gyrus (IFG). Channel assignment to the different ROIs was determined following Rorden and Brett (2000); Singh et al. (2005), and Tsuzuki et al. (2007).

### fNIRS Connectivity Analyses

Furthermore, functional connectivity (FC) was computed by Pearson correlations after correcting for outliers for the data of each channel pair. Correlation coefficients were normalized by Fisher’s r-to-z transformation. The analysis strategy proposed by Zhu et al. (2017) was applied: FC was compared within the predefined ROIs (average correlation of all channels within the ROI), between the ROIs and the other brain areas covered by the probe set (average correlation between the channels of the ROI and the channels of a given brain area) (Zhu et al., 2017). Correction for multiple comparisons was performed using the procedure of Armitage-Parmar at a significance level of α = 0.05 (Sankoh et al., 1997). This correction method was chosen due to the high intercorrelation of the different NIRS channels.

### Statistical Processing: Stroop Data

All analyses of the Stroop data were run with SPSS 22 (SPSS Inc., Chicago, United States), and the inverse efficiency score $[IES=\frac{RT}{1-Proportionoferrors}$; Townsend and Ashby (1983)] was used (reaction time = RT). Outlier trials (more than 2 standard deviations from the mean per subject, in total 3.72% of the data) and incorrect trials were excluded from the analyses. To test the presence of an emotional Stroop effect (and corresponding influences of the independent variables), a 2 × 2 × 3 repeated-measures ANOVA was conducted. Between-participants factors were cognitive control (high- vs. low-impulsive group) and instruction before emotion induction (suppress vs. allow arising emotions); within-participants factor was stimulus valence (neutral vs. negative vs. positive). As a *post hoc* analysis, further one-way ANOVAs were conducted with IES as dependent variable and instruction as single factor for each group separately.

### Statistical Processing: Correlations

Correlations with global connectivity, EMG values for time segment 11 (most arousing sequence of the movie clip and highest activation over all participants), and the overall IES score in the Stroop task were calculated. Pearson’s method was used separately for each group and condition (low- vs. high-impulsive, suppress vs. allow). For multiple testing, a Bonferroni-Holm correction was applied (Holm, 1979). The IES score and the global connectivity were approximately normally distributed, as assessed by the Shapiro-Wilk-Test, *p* < 0.05. The EMG values were not normally distributed, according to the Shapiro-Wilk-Test. Therefore, we used the following formula: $\sqrt{EMG}\times -1$ for normalizing the EMG results (cf. Osborne, 2002). After this correction the data met the criteria for normal distribution (Shapiro-Wilk-Test, *p* < 0.05).

## Results

### Emotion Induction—EMG Results

**FIGURE 2:**
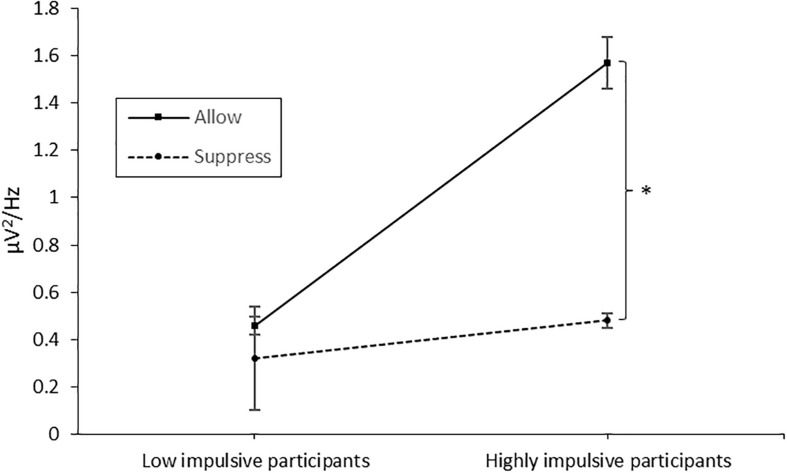
Muscle activity over the corrugator supercilii in the *allow*ance and *suppression* condition, separated for the low- and high-impulsive groups. Error bars indicate the standard error. The star denotes the significant difference between the conditions in the high-impulsive group. The asterisk marks the significant difference.

The 2 × 2 × 12 ANOVA (group × instruction × time) revealed a significant main effect of time [*F*_(__11,43)_ = 16.864, *p* < 0.001, η^2^ = 0.282], a main effect of group [*F*_(__11,43)_ = 7.266, *p* = 0.010, η^2^ = 0.145; *M*_LowImpulsive_ = 0.40 μV^2^/Hz, *M*_HighlyImpulsive_ = 1.03 μV^2^/Hz], a main effect of instruction [*F*_(__11,43)_ = 6.863, *p* = 0.012, η^2^ = 0.138; *M*_Suppress_ = 0.40 μV^2^/Hz, *M*_Allow_ = 1.02 μV^2^/Hz], an interaction effect of time and group [*F*_(__11,43)_ = 4.358, *p* < 0.001, η^2^ = 0.092], an interaction effect of instruction and time [*F*_(__11,43)_ = 2.87, *p* = 0.001, η^2^ = 0.062] and an interaction effect of group and instruction [*F*_(__11,43)_ = 4.008, *p* = 0.049, η^2^ = 0.087]. As the interaction of group and instruction is directly related to our hypotheses, we performed a *post hoc* test, merged the data for time, and separately ran a *t*-test for paired measurements for each group. In accordance with our hypotheses, no significant difference between conditions (instruction allow vs. suppress) was found in the low-impulsive group [*t*(20) = 0.872, *p* = 0.393], whereas for the high-impulsive participants [*t*(23) = 2.623, *p* = 0.015], muscle activity differed significantly between instructions with higher values in the instruction condition allow (1.57 μV^2^/Hz vs. 0.48 μV^2^/Hz; see Figure 2).

### Emotion Induction—Connectivity Results

**FIGURE 3:**
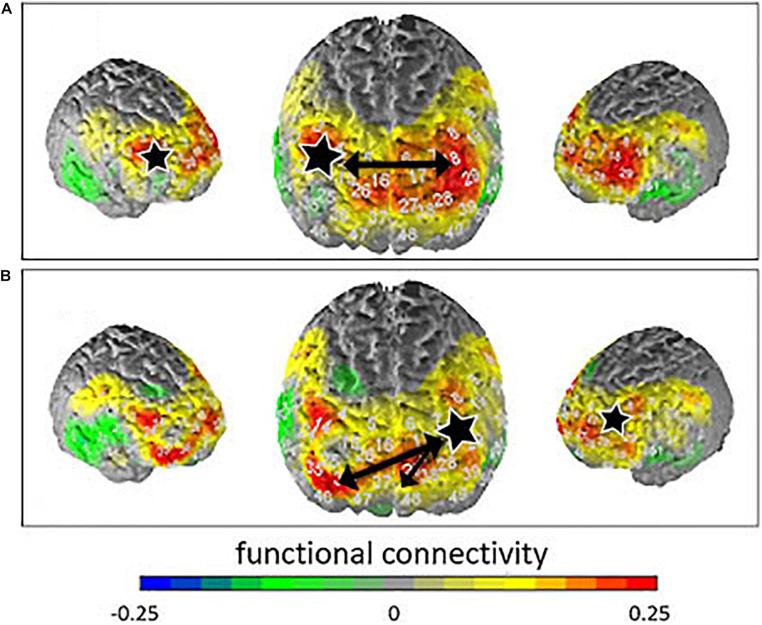
Contrasted connectivity (instruction *suppress—*instruction *allow*) for the seed regions (marked with a white star) right DLPFC **(A)** and left frontopolar area **(B)**. Functional connectivity is indicated by the different colors. The black arrows indicate significant correlations.

No interaction effects and no group effects were found in the connectivity analyses. After Armitage-Parmar correction for multiple testing, the correlations between the right DLPFC (BA46) and the left DLPFC (BA46; *p* = 0.0226, *r* = 0.49) as well as the correlations between the left DLPFC (BA46) and the right and left frontopolar areas (BA10; *p* = 0.0352, *r* = 0.53; *p* = 0.0135, *r* = 0.65) remained significant. Thus, significantly stronger connectivity was observed between these different response regions for the *suppress all arising emotion condition* compared to the *allow all arising emotion* condition. Figure 3 shows the contrast of the correlation for suppression minus allowance instruction.

### Stroop Task—Behavioral Results

Statistical analysis (2 × 2 × 3 repeated-measures ANOVA with mixed factors group, instruction, and stimulus valence) showed no significant effect of stimulus valence on behavioral data. Neither the main effect [*F*_(__2,__114)_ = 0.36, *p* = 0.701] nor interactions with other factors [with group: *F*_(__2,__114)_ = 1.88, *p* = 0.157; with instruction: *F*_(__2,__114)_ = 1.51, *p* = 0.226; with group and instruction: *F*_(__2,__114)_ = 1.64, *p* = 0.198] were significant (see Supplementary Figure 1). Stimulus valence did not influence response speed or correctness in any case (see Supplementary Figure 2). Therefore, the stimulus valence was merged, and the results of the Stroop task were used as a general measurement of cognitive control capability. The RTs, proportion of errors and IES are listed in Table 2.

**TABLE 2:** Mean RTs (in ms), mean error rates (in percent), and mean IES (in ms) for all trials of all factor-level combinations (SD in brackets).

	Low-impulsive group (*N* = 25)		High-impulsive group (*N* = 32)	
	Suppress (*N* = 13)	Allow (*N* = 12)	Suppress (*N* = 16)	Allow (*N* = 16)
Mean RT	568 (150)	572 (135)	600 (139)	619 (153)
Mean Error rates (ER)	11.7 (5.8)	11.5 (7.2)	13.3 (6.6)	17.0 (8.8)
Mean IES	647 (180)	651 (166)	700 (186)	758 (218)

Analysis revealed a significant main effect of group on IES [*F*_(__1,57)_ = 8.92, *p* = 0.004, η^2^ = 0.013]. Participants in the low-impulsive group (*M* = 649 ms) achieved a significantly smaller (better) mean IES [*t*(57) = −2.99, *p* = 0.004] than participants of the high-impulsive group (*M* = 737 ms). However, neither a significant main effect of instruction on IES [*F*_(__1,__57)_ = 1.62, *p* = 0.209, η^2^ = 0.02] nor a significant interaction between group and instruction [*F*_(__1,__57)_ = 1.14, *p* = 0.289, η^2^ = 0.02] could be found.

### Correlational Results

In the low-impulsive group with the instruction to allow all arising emotions, we found a significant correlation between global connectivity and the IES [*r*(9) = 0.712, *p* = 0.014] as well as between EMG activation and IES [*r*(9) = 0.721, *p* = 0.012]. In both cases, higher connectivity and EMG activation were associated with higher (worse) IES. In the other group and conditions, no correlation was significant (see all correlations in Table 3).

**TABLE 3:** Correlations between EMG, connectivity and IES for the different groups and conditions.

Group	Instruction	$\sqrt{\text{EMG}}$ × –1 – Connectivity	Connectivity – IES	$\sqrt{\text{EMG}}$ × –1 – IES
Low-impulsive	Allow (*N* = 11)	*r*(9) = 0.405, *p* = 0.217	*r*(9) = 0.712, *p* = 0.014*	*r*(9) = −0.679, *p* = 0.021*
	Suppress (*N* = 10)	*r*(8) = −0.453, *p* = 0.188	*r*(8) = −0.464, *p* = 0.151	*r*(8) = −0.045, *p* = 0.901
High- impulsive	Allow (*N* = 13)	*r*(11) = −0.230, *p* = 0.449	*r*(11) = −0.277, *p* = 0.360	*r*(11) = −0.045, *p* = 0.884
	Suppress (*N* = 12)	*r*(10) = −0.120, *p* = 0.710	*r*(10) = 0.134, *p* = 0.678	*r*(10) = −0.446, *p* = 0.147

Scatterplots of the significant correlations are depicted in Supplementary Material. *Significant for Bonferroni-Holm corrected α.

## Discussion

The present study aimed to investigate the effects of emotion regulation (vs. no emotion regulation) in high- vs. low-impulsive participants and the underlying functional connectivity within the CCN. In line with our hypotheses, we found a significant effect of impulsivity (group) on both EMG activation during emotion induction and subsequent Stroop performance. High-impulsive participants, independent of instruction, showed higher EMG activation and worse performance in the emotional Stroop task. No group effect was found for connectivity of the DLPFC during emotion induction. Main effects of instruction were found for EMG activation—with significantly higher values for the allowance instruction—and for connectivity analyses—with significantly higher correlations between the right and left DLPFC (BA46) as well as the left DLPFC and the right and left frontopolar area (BA10) in the suppression condition compared to the allowance condition. No significant difference was found for the emotional Stroop task performance between instruction conditions. As hypothesized, an interaction effect of group and instruction was found for EMG activation, with a significant difference between instruction conditions only for high-impulsive participants. Such an interaction effect was not found for the connectivity analysis and the emotional Stroop task. Significant correlations between IES and global connectivity as well as EMG activity were seen for low-impulsive participants in the allowance condition.

**FIGURE 4:**
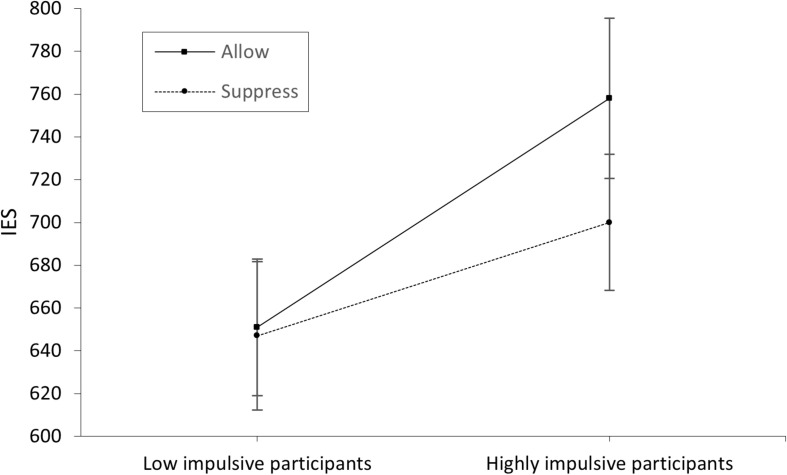
IES scores of the low- and high-impulsive group separated by allowance and suppression instruction. The error bars indicate the standard error.

The significant main effect of instruction in the EMG data shows the correct effect of our manipulation. This confirms the correlation between negative or unpleasant emotions and the corresponding facial expression (e.g., Larsen et al., 2003) indicated by the activation of the corrugator supercilii, which is well described in the literature (e.g., Cacioppo et al., 1986; Lang et al., 1993). The overall higher EMG activation during the emotion induction paradigm in high-impulsive participants might suggest that successful emotion regulation requires cognitive control. However, given that no active manipulation of impulsivity occurred as an independent variable, the data rather indicate an undirected, close association between emotion regulation and cognitive control. The significant interaction effect found for group and instruction for EMG activation during emotion induction illustrates the expected ceiling effect in low-impulsive participants. While persons with high cognitive control seem to implicitly regulate their emotions independently of external stimuli (such as the instruction to suppress vs. allow), persons with low cognitive control might require external cues (in this study, the instruction to suppress arising emotions) to regulate their emotions to the same extent as low impulsive participants. This result indicates the efficiency and usefulness of instructions, as used in cognitive behavioral therapy, to regulate negative emotions. While in the low-impulsive group (i.e., high cognitive control) a ceiling effect seemed to limit the impact of emotion regulation instructions, this external stimulus had an effect in the high-impulsive (i.e., low cognitive control) group. The relationship of explicit and implicit emotion regulation with cognitive control mechanisms has been well described (cf. Egner et al., 2007; Gyurak et al., 2011). Strengthening cognitive control can potentially be considered as a general therapeutic approach. In this context, Wolkenstein and Plewnia (2013) successfully examined neuromodulation (transcranial direct current stimulation of the DLPFC) as a method to enhance cognitive control in a depressive sample. Since Vanderhasselt and De Raedt (2009) found a relation between improved cognitive control and fewer depressive episodes, such approaches seem promising for clinical application (Siegle et al., 2007). Nevertheless, we found no group effect in functional connectivity between the brain areas investigated. Although there are several possible explanations, such as insufficient sample size, this finding might suggest that low-impulsive participants achieved better control over their emotions than high-impulsive participants with the same level of connectivity. This would suggest a more efficient use of frontal brain networks in the low-impulsive group. Alternatively, group differences in EMG activation and subsequent Stroop performance could be related to differences in brain areas not measured by our fNIRS probe set (e.g., the ACC; Koski and Paus, 2000). The increased connectivity during active emotion regulation, especially between BA46/DLPFC and other frontal lobe areas, could be interpreted as confirmation of the CCN model (Koski and Paus, 2000; Ridderinkhof et al., 2004) and the need for cognitive control for effective emotion regulation. It is also in accordance with the model of Ochsner and Gross (2005), which allocates reappraisal processes (cf. instruction conditions) primarily to the DLPFC. That these instructional effects (of suppressing vs. allowing arising emotions) did not impact behavioral data in the emotional Stroop task could be due to the delay between the emotion induction and subsequent task performance. This delay of approximately 5 min, together with a limited number of participants in the different groups, could have decreased the effects to no significance, as a numerical difference is noticeable (at least in the high-impulsive group; see Figure 4). Another limiting factor could be that participants were not asked about what strategy they applied to suppress their emotional reaction. Differences regarding this would have been a relevant variable to control for. Conceivably, high-impulsive participants were more likely to use maladaptive regulatory strategies that were only temporarily successful, attenuating an instructional effect over time (Mitchell et al., 2012; cf. Schreiber et al., 2012). Another limitation is the gender imbalance between men and women (48 female vs. 9 male). It is known that behaviorally there are only small differences in emotion regulation between men and women. But there are significant neuronal differences, so that in future studies attention should be paid to a balanced sex ratio to be able to investigate these differences in more detail (cf. McRae et al., 2008).

Considering the involvement of BA46 in particular in the regulation of negative emotions, a specialized therapeutic approach, comparable to that of Wolkenstein and Plewnia (2013), could be developed using neuromodulation to treat patients with clinically relevant emotion regulation problems. Thus, future studies should examine the effect of transcranial direct current or transcranial magnetic stimulation of the DLPFC—or more specifically BA46—on emotion regulation in participants with reduced cognitive control. Causal conclusions regarding the involvement of the DLPFC in emotion regulation would also be possible if the current study design were combined with neuromodulation. With a lager sample considering the four experimental groups of the study design, effects of gender and handedness could also be investigated in future studies. Another interesting study design would be to combine the present study paradigm with neurofeedback. Since neurofeedback is a very effective treatment tool (cf. Barth et al., 2016), it is reasonable to assume that it would also influence emotion regulation skills.

The interpretation of correlational results should be taken with caution due to the limited number of participants, several outliers –which could have decisive influence on the results (see Supplementary Figures 4,5) –and lack of normality in each group. Therefore, even after correction for multiple testing, it is difficult to assume reliability of the results despite their significance because of the factors mentioned above. Nevertheless, significant correlations between global connectivity and EMG activation with higher (= worse) IES in the low-impulsive group (allowance condition only) suggest a connection between participants who need more frontal connectivity to (implicitly) control themselves during negative emotion induction and reduced performance in the emotional Stroop task. Significant correlations only in this subgroup could be explained by the high variability in this group (high cognitive resources and no forced emotion regulation). Differences in task performance associated with global connectivity might not have been detected for other subgroups, since the level of cognitive control was consistently high (low impulsivity and forced emotion regulation) or low (high impulsivity).

Considering self-control abilities and their neural correlates (e.g., frontal connectivity) as a limited resource, it seems necessary to also consider possible depletion effects as found by Niven et al. (2013) when interpreting the current results. In their study, blood glucose levels decreased following emotion regulation in participants with (perceived) low self-control. Although blood glucose levels are a general measure, previous findings support the conceptualization of glucose as a limited energy resource on which self-control abilities rely, along with others (e.g., Gailliot et al., 2007). In the current study, however, Stroop task performance was not negatively affected by preceding emotion regulation. This could be interpreted to mean that experiencing negative emotions affects cognitive control more than potential depletion of self-control abilities resulting from emotion regulation. This seems to be especially the case for participants with *a priori* low cognitive control resources.

Also important to keep in mind when interpreting the results is the conceptualization and operationalization of impulsivity and impulsive behavior as used in the current study. Recent studies by Herman et al. (2018) and Johnson et al. (2020) suggest a more nuanced view of impulsivity, e.g., distinguishing between impulsive responses to negative vs. positive emotions or between different aspects of impulsivity (state vs. trait, attentional vs. motor processes). Future research examining emotion regulation and cognitive control in association with impulsivity should take these differentiations into account.

Based on this study, we conclude that the CCN is closely associated with emotion regulation capabilities. BA46 seems to play a crucial role. While persons with high cognitive control show implicit capabilities to regulate their emotions during a negative emotion induction independent of external instructions, persons with low cognitive control capabilities need external instructions (= explicit emotion regulation) to achieve similarly low expressions of emotionality.

## Data Availability Statement

The raw data supporting the conclusions of this article will be made available by the authors, without undue reservation.

## Ethics Statement

The studies involving human participants were reviewed and approved by the Ethics Comittee of the University Hospital Tübingen. The patients/participants provided their written informed consent to participate in this study.

## Author Contributions

MM, JS, and A-CE contributed to the conception and design of the study. MM and JS ran the study, organized the data, and wrote the first draft of the manuscript. MM, JS, DR, and A-CE performed the statistical analysis. A-CE, DR, MH, and AF wrote sections of the manuscript. All authors critically revised the manuscript for important intellectual content and read and approved the submitted version.

## Conflict of Interest

The authors declare that the research was conducted in the absence of any commercial or financial relationships that could be construed as a potential conflict of interest.

## Publisher’s Note

All claims expressed in this article are solely those of the authors and do not necessarily represent those of their affiliated organizations, or those of the publisher, the editors and the reviewers. Any product that may be evaluated in this article, or claim that may be made by its manufacturer, is not guaranteed or endorsed by the publisher.

## Supplementary Material

Supplementary Figure 1 Mean IES (in ms) for all trials as a function of group (low- vs. high-impulsive), instruction (allow vs. suppress arising emotions) and stimulus valence (neutral vs. negative vs. positive). Gray dotted lines: Mean IES across all stimulus valences. For this graph, IES formula was applied with stimulus valence-specific ERs.

Supplementary Figure 2 Boxplots of valence-dependent reaction times and error rates (Neu, neutral; Pos, positive; Neg, negative) as well as *p*-values of paired *t*-tests comparing different valences.

Supplementary Figure 3 Schematic depiction of the button box setup: All four buttons were pushed with the left or right middle or index finger. The color of the buttons is supposed to indicate the button-color assignment—it does not represent the actual color of the button material, which was white.

Supplementary Figure 4 Scatterplot of the significant correlation between Connectivity and Mean IES in the low impulsive group in the condition “allow all upcoming feelings.”

Supplementary Figure 5 Scatterplot of the significant correlation between EMG and Mean IES in the low impulsive group in the condition “allow all upcoming feelings.”

Supplementary Table 1 Mean experienced anger, fear and sadness during the emotion induction paradigm depending on group and instruction (SD in brackets).
