# Supplementary material for: To Regulate or Not to Regulate: Emotion Regulation in Participants With Low and High Impulsivity
Source: Front Behav Neurosci. 2021 Jul 30;15:645052. doi: 10.3389/fnbeh.2021.645052 (PMC8363082; doi:10.3389/fnbeh.2021.645052)
Supplement: Supplementary Figure 1 — Mean IES (in ms) for all trials as a function of group (low- vs. high-impulsive), instruction (allow vs. suppress arising emotions) and stimulus valence (neutral vs. negative vs. positive). Gray dotted lines: Mean IES across all stimulus valences. For this graph, IES formula was applied with stimulus valence-specific ERs. [file Data_Sheet_1.pdf]

Appendix

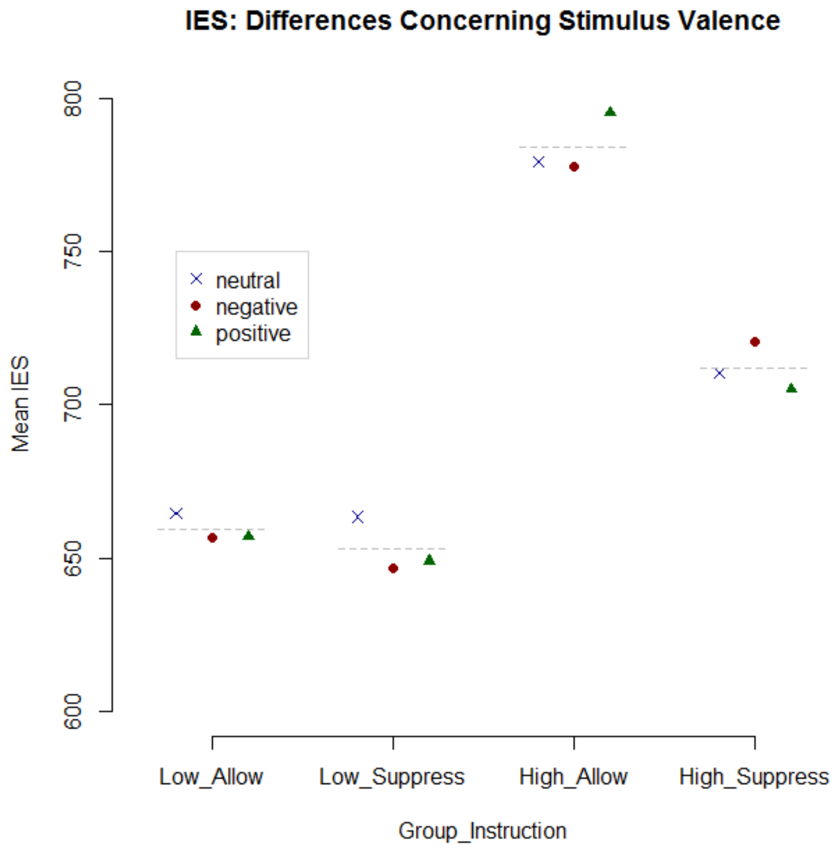

Figure S1. Mean IES (in ms) for all trials as a function of group (low- vs. high-impulsive), instruction (allow vs. suppress arising emotions) and stimulus valence (neutral vs. negative vs. positive). Grey dotted lines: Mean IES across all stimulus valences. For this graph, IES formula was applied with stimulus valence-specific ERs.

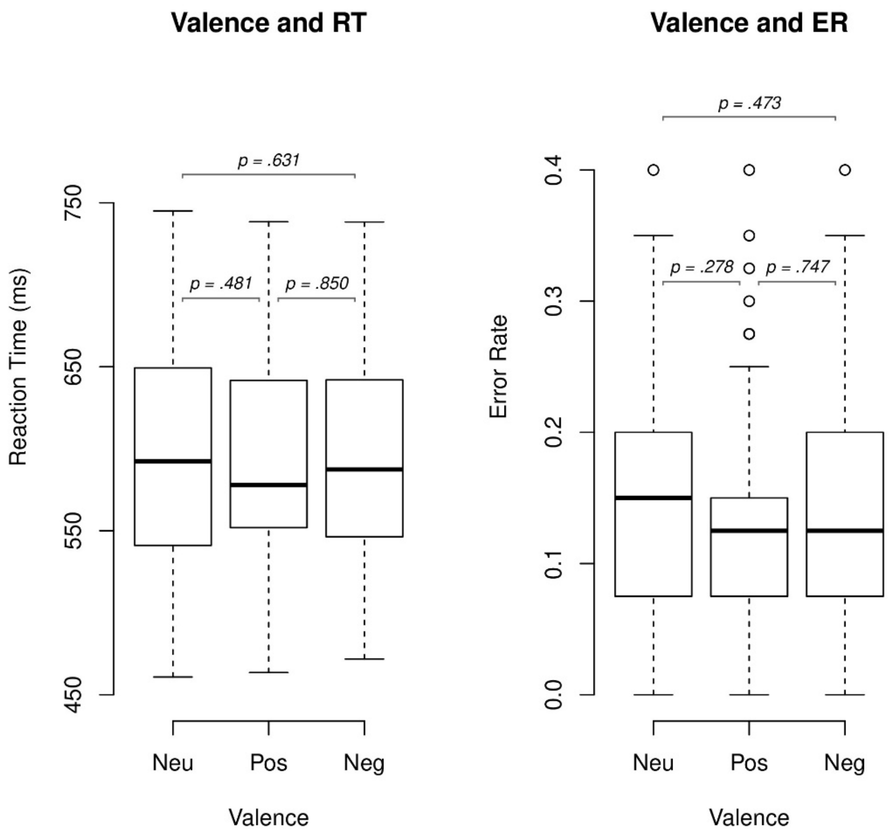

Figure S2. Boxplots of valence-dependent reaction times and error rates (Neu = neutral, Pos = positive, Neg = negative) as well as *p*-values of paired t-tests comparing different valences.

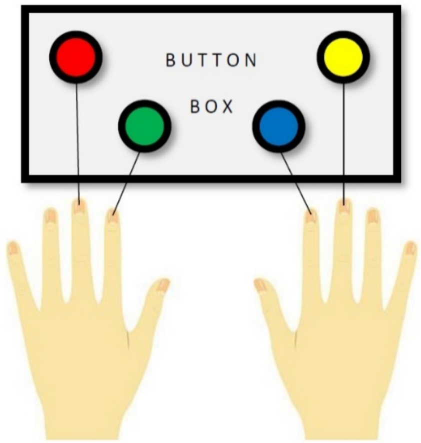

Figure S3. Schematic depiction of the button box setup: All four buttons were pushed with the left or right middle or index finger. The color of the buttons is supposed to indicate the button-color assignment – it does not represent the actual color of the button material, which was white.

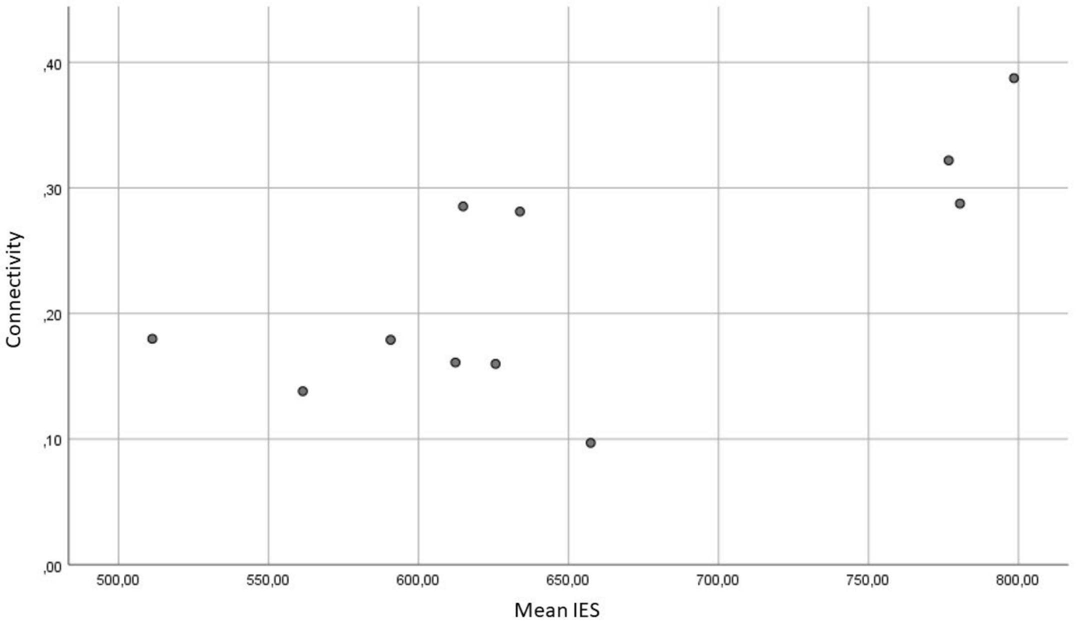

Figure S4: Scatterplot of the significant correlation between Connectivity and Mean IES in the low impulsive group in the condition “allow all upcoming feelings”.

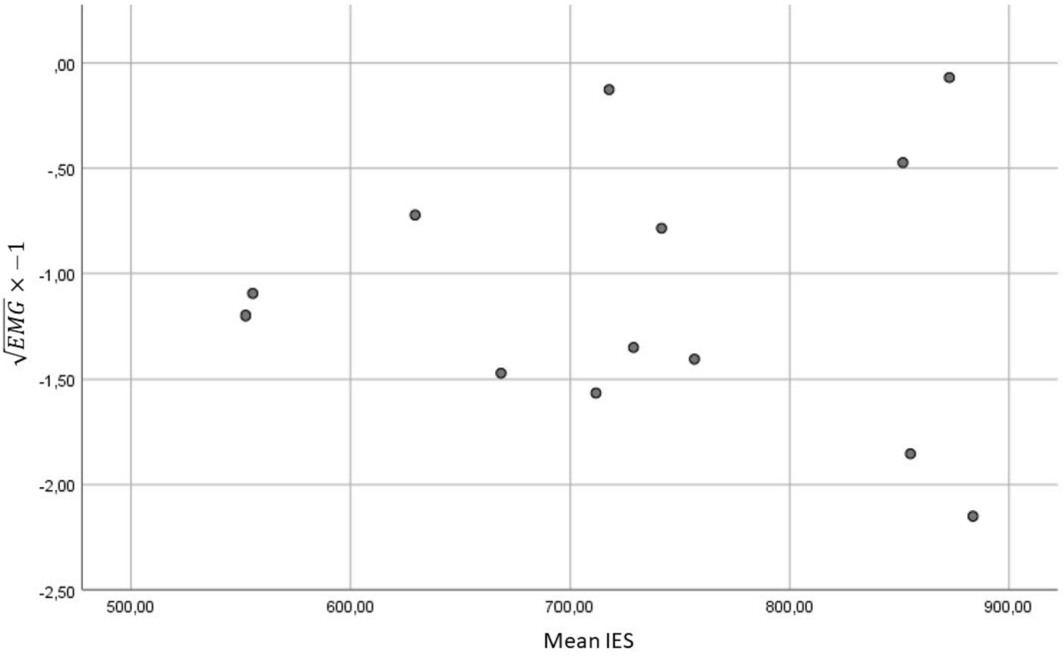

Figure S5: Scatterplot of the significant correlation between EMG and Mean IES in the low impulsive group in the condition “allow all upcoming feelings”.

Table S1

Mean experienced anger, fear and sadness during the emotion induction paradigm depending on group and instruction (SD in brackets).

Group		Anger	Fear	Sadness
Low-impulsive	Allow (*N* = 10)	3.80 (0.92)	2.7 (1.49)	3.80 (1.23)
	Suppress (*N* = 12)	3.00 (1.21)	2.33 (1.15)	3.83 (0.94)
High-impulsive	Allow (*N* = 14)	3.57 (1.16)	2.07 (1.14)	3.71 (0.91)
	Suppress (*N* = 17)	3.35 (0.79)	2.17 (0.95)	3.76 (0.83)

Note. Participants had to report if they experienced anger, fear or sadness while watching the clip: (1) “very little”, (2) “little”, (3) moderately, (4) “much”, (5) “very much"
